# Supplementary material for: An Evaluation Benchmark for Adverse Drug Event Prediction from Clinical Trial Results
Source: Sci Data. 2025 Mar 11;12:424. doi: 10.1038/s41597-025-04718-1 (PMC11897206; doi:10.1038/s41597-025-04718-1)
Supplement: Supplementary file 1 — Supplementary information [file 41597_2025_4718_MOESM1_ESM.docx]

**Supplementary information**

**An Evaluation Benchmark for Adverse Drug Event Prediction from Clinical Trial Results**

Anthony Yazdani^1,*^, Alban Bornet^1^, Philipp Khlebnikov^2^, Boya Zhang^1^, Hossein Rouhizadeh^1^, Poorya Amini^2^ and Douglas Teodoro^1,*^

^1^Department of Radiology and Medical Informatics, Faculty of Medicine, University of Geneva, Geneva, Switzerland

^2^Risklick AG, Bern, Switzerland

^*^corresponding author(s): [anthony.yazdani@unige.ch](mailto:anthony.yazdani@unige.ch), [douglas.teodoro@unige.ch](mailto:douglas.teodoro@unige.ch)


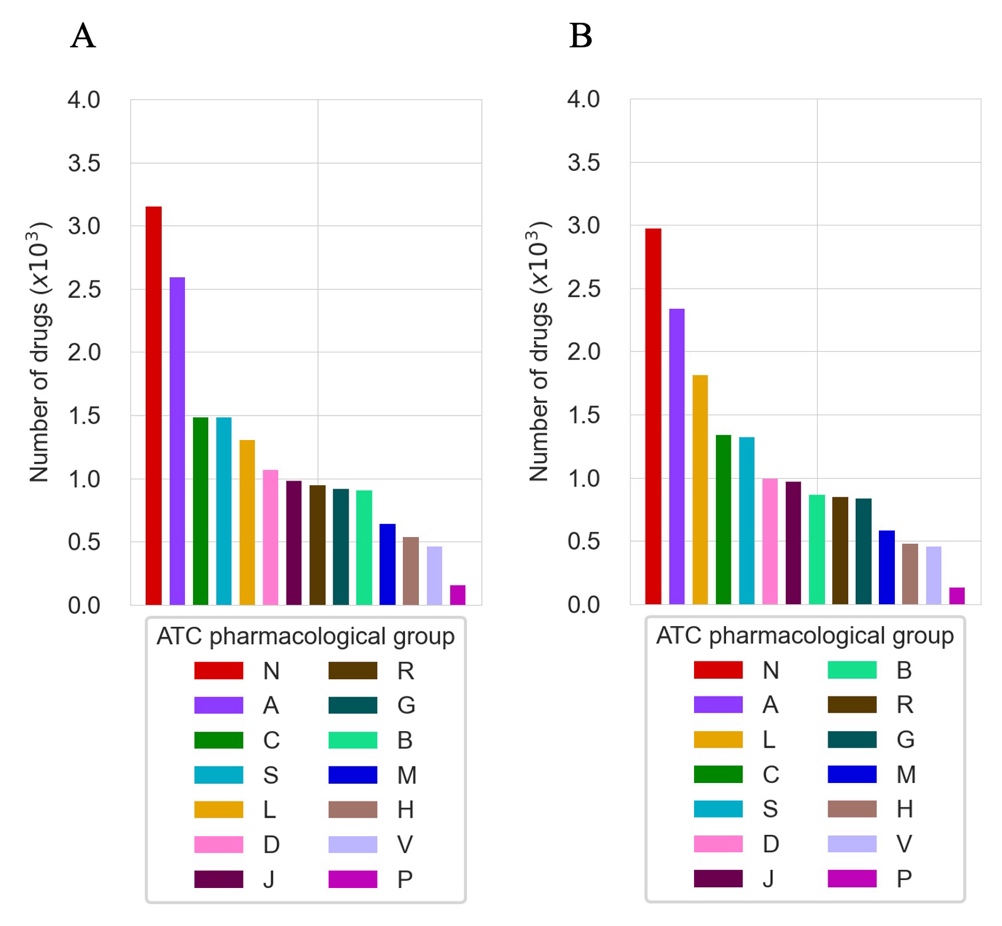


Supplementary Figure 1. (A) Representation of ATC main pharmacological groups in CT-ADE-SOC. (B) Representation of ATC main pharmacological groups in CT-ADE-PT.

| **MedDRA Level** | **Model** | **Precision (%)** | **Recall (%)** | **F1-score (%)** | **Accuracy (%)** | **Balanced Accuracy (%)** | **AUROC (%)** |
| --- | --- | --- | --- | --- | --- | --- | --- |
| PT | MAJ | 0.00 | 0.00 | 0.00 | **99.95** | 50.00 | - |
|  | S | 0.09 | 2.78 | 0.17 | 98.32 | 50.57 | 73.18 |
|  | SG | 48.59 | 24.50 | 32.58 | **99.95** | 62.25 | 98.04 |
|  | SGE | **49.63** | **31.29** | **38.38** | **99.95** | **65.64** | **98.23** |

Supplementary Table 1. Performance of discriminative models on the CT-ADE-PT test set. MAJ: Majority class prediction; S: SMILES; SG: SMILES and group description; SGE: SMILES, group description, and eligibility criteria. Results are micro-averaged.

| **MedDRA Level** | **Comparison** | **Full test set** | **Positive subset** |
| --- | --- | --- | --- |
| PT | S vs. SG | < .001 | < .001 |
|  | S vs. SGE | < .001 | < .001 |
|  | SG vs. SGE | 0.05 | < .001 |

Supplementary Table 2. P-values from pairwise McNemar's tests comparing the performance of discriminative models on the CT-ADE-PT test set using different feature sets. The tests were conducted on the entire test set (Full test set) and the subset of the test set where ADEs were observed (Positive subset). S: SMILES; SG: SMILES and group description; SGE: SMILES, group description, and eligibility criteria.

| **Type** | **Backbone** | **Features** | **Precision (%)** | **Recall**  **(%)** | **F1-score (%)** | **Accuracy (%)** | **Balanced Accuracy (%)** | **AUROC (%)** |
| --- | --- | --- | --- | --- | --- | --- | --- | --- |
| MAJ | - | - | 0.00 | 0.00 | 0.00 | 88.68 | 50.00 | - |
| Discriminative | ChemBERTa and PubMedBERT | S | 42.65 | 25.56 | 31.96 | 87.69 | 60.59 | 79.90 |
|  |  | SG | 51.07 | 42.00 | 46.09 | 88.88 | 68.43 | 84.92 |
|  |  | SGE | 51.65 | 55.40 | **53.46** | 89.08 | **74.39** | **88.34** |
| Generative | OpenBioLLM-8B | S | 44.55 | 16.57 | 24.16 | 88.22 | 56.97 | - |
|  |  | SG | 49.14 | 50.36 | 49.74 | 88.48 | 71.86 | - |
|  |  | SGE | **52.18** | 54.75 | 53.43 | **89.20** | 74.17 | - |

Supplementary Table 3. Performance of ChemBERTa & PubMedBERT, and OpenBioLLM-8B on the CT-ADE-SOC test set using S, SG, and SGE feature sets. The AUROC metric cannot be computed for baseline and generative models because these models do not produce raw probabilities. MAJ: Majority class prediction; S: SMILES; SG: SMILES and group description; SGE: SMILES, group description, and eligibility criteria. Results are micro-averaged.

| **Abbreviation** | **System organ class (SOC)** |
| --- | --- |
| Blood | Blood and lymphatic system disorders |
| Card | Cardiac disorders |
| Cong | Congenital, familial and genetic disorders |
| Ear | Ear and labyrinth disorders |
| Endo | Endocrine disorders |
| Eye | Eye disorders |
| Gastr | Gastrointestinal disorders |
| Genrl | General disorders and administration site conditions |
| Hepat | Hepatobiliary disorders |
| Immun | Immune system disorders |
| Infec | Infections and infestations |
| Inj&P | Injury, poisoning and procedural complications |
| Inv | Investigations |
| Metab | Metabolism and nutrition disorders |
| Musc | Musculoskeletal and connective tissue disorders |
| Neopl | Neoplasms benign, malignant and unspecified (incl cysts and polyps) |
| Nerv | Nervous system disorders |
| Preg | Pregnancy, puerperium and perinatal conditions |
| Psych | Psychiatric disorders |
| Renal | Renal and urinary disorders |
| Repro | Reproductive system and breast disorders |
| Resp | Respiratory, thoracic and mediastinal disorders |
| Skin | Skin and subcutaneous tissue disorders |
| SocCi | Social circumstances |
| Surg | Surgical and medical procedures |
| Vasc | Vascular disorders |
| Prod | Product issues |

Supplementary Table 4. Mapping of system organ class (SOC) abbreviations to their full terms.

| **Abbreviation** | **ATC full term** |
| --- | --- |
| A | Alimentary tract and metabolism |
| B | Blood and blood forming organs |
| C | Cardiovascular system |
| D | Dermatologicals |
| G | Genito urinary system and sex hormones |
| H | Systemic hormonal preparations, excl. sex hormones and insulins |
| J | Antiinfectives for systemic use |
| L | Antineoplastic and immunomodulating agents |
| M | Musculo-skeletal system |
| N | Nervous system |
| P | Antiparasitic products, insecticides and repellents |
| R | Respiratory system |
| S | Sensory organs |
| V | Various |

Supplementary Table 5. Mapping of anatomical therapeutic chemical (ATC) main pharmacological group abbreviations to their full terms.

| **Label** | **Precision (%)** | **Recall (%)** | **F1-score (%)** | **Accuracy (%)** | **Balanced Accuracy (%)** | **AUROC (%)** |
| --- | --- | --- | --- | --- | --- | --- |
| Blood and lymphatic system disorders | 56.36 | 44.29 | 49.60 | 90.00 | 70.00 | 87.45 |
| Cardiac disorders | 27.03 | 37.74 | 31.50 | 86.19 | 64.19 | 75.03 |
| Congenital, familial and genetic disorders | 0.00 | 0.00 | 0.00 | 99.76 | 50.00 | 70.38 |
| Ear and labyrinth disorders | 26.67 | 10.00 | 14.55 | 96.27 | 54.55 | 82.77 |
| Endocrine disorders | 37.50 | 13.04 | 19.35 | 98.02 | 56.32 | 70.68 |
| Eye disorders | 43.48 | 27.03 | 33.33 | 90.48 | 61.82 | 80.39 |
| Gastrointestinal disorders | 62.37 | 84.99 | 71.95 | 73.02 | 74.89 | 82.41 |
| General disorders and administration site conditions | 57.86 | 60.07 | 58.94 | 80.87 | 73.55 | 83.43 |
| Hepatobiliary disorders | 11.11 | 3.85 | 5.71 | 97.38 | 51.60 | 78.35 |
| Immune system disorders | 100.00 | 5.56 | 10.53 | 98.65 | 52.78 | 63.66 |
| Infections and infestations | 51.01 | 75.75 | 60.96 | 71.75 | 72.93 | 80.77 |
| Injury, poisoning and procedural complications | 25.93 | 19.81 | 22.46 | 88.49 | 57.31 | 74.28 |
| Investigations | 48.94 | 59.15 | 53.56 | 80.87 | 72.50 | 81.64 |
| Metabolism and nutrition disorders | 34.68 | 54.09 | 42.26 | 81.35 | 69.69 | 81.66 |
| Musculoskeletal and connective tissue disorders | 43.10 | 55.41 | 48.48 | 78.41 | 69.49 | 79.99 |
| Neoplasms benign, malignant and unspecified | 0.00 | 0.00 | 0.00 | 97.62 | 49.96 | 88.40 |
| Nervous system disorders | 64.35 | 79.89 | 71.28 | 73.33 | 74.29 | 82.94 |
| Pregnancy, puerperium and perinatal conditions | 75.00 | 42.86 | 54.55 | 99.60 | 71.39 | 90.71 |
| Product issues | 0.00 | 0.00 | 0.00 | 99.44 | 49.92 | 75.00 |
| Psychiatric disorders | 69.41 | 32.07 | 43.87 | 88.02 | 64.82 | 88.32 |
| Renal and urinary disorders | 40.00 | 14.29 | 21.05 | 91.67 | 56.24 | 77.71 |
| Reproductive system and breast disorders | 44.44 | 8.16 | 13.79 | 96.03 | 53.88 | 74.66 |
| Respiratory, thoracic and mediastinal disorders | 47.09 | 36.32 | 41.01 | 81.51 | 63.77 | 76.32 |
| Skin and subcutaneous tissue disorders | 42.93 | 43.39 | 43.16 | 82.86 | 66.60 | 77.88 |
| Social circumstances | 0.00 | 0.00 | 0.00 | 99.92 | 50.00 | 66.44 |
| Surgical and medical procedures | 44.44 | 26.67 | 33.33 | 98.73 | 63.13 | 69.81 |
| Vascular disorders | 41.61 | 41.36 | 41.49 | 85.00 | 66.40 | 79.96 |

Supplementary Table 6. Discriminative SGE performance metrics per label on CT-ADE-SOC test set.

| **ATC Category** | **Precision (%)** | **Recall (%)** | **F1-score (%)** | **Accuracy (%)** | **Balanced Accuracy (%)** | **AUROC (%)** |
| --- | --- | --- | --- | --- | --- | --- |
| Alimentary Tract and Metabolism | 40.97 | 46.97 | 43.76 | 92.43 | 71.22 | 88.61 |
| Blood and Blood Forming Organs | 45.71 | 69.57 | 55.17 | 88.67 | 80.18 | 86.81 |
| Cardiovascular System | 49.41 | 41.18 | 44.92 | 92.05 | 68.79 | 87.27 |
| Dermatologicals | 47.66 | 43.88 | 45.69 | 92.54 | 70.09 | 88.82 |
| Genito Urinary System and Sex Hormones | 42.67 | 40.51 | 41.56 | 95.67 | 69.18 | 89.81 |
| Systemic Hormonal Preparations | 48.00 | 75.00 | 58.54 | 90.31 | 83.42 | 90.59 |
| Antiinfectives for Systemic Use | 26.77 | 40.96 | 32.38 | 90.44 | 67.17 | 86.59 |
| Antineoplastic and Immunomodulating Agents | 57.35 | 57.88 | 57.62 | 79.85 | 72.27 | 82.76 |
| Musculo-Skeletal System | 41.86 | 44.63 | 43.20 | 90.77 | 69.67 | 87.73 |
| Nervous System | 56.08 | 59.72 | 57.84 | 91.57 | 77.35 | 90.66 |
| Antiparasitic Products, Insecticides and Repellents | 38.46 | 45.98 | 41.88 | 90.86 | 70.15 | 87.46 |
| Respiratory System | 51.95 | 47.62 | 49.69 | 94.12 | 72.38 | 90.73 |
| Sensory Organs | 60.87 | 50.00 | 54.90 | 97.63 | 74.52 | 92.76 |
| Various | 49.47 | 55.95 | 52.51 | 92.32 | 75.63 | 87.47 |
| No ATC | 52.96 | 57.20 | 55.00 | 87.58 | 74.71 | 87.72 |

Supplementary Table 7. Discriminative SGE performance metrics by ATC main pharmacological groups on the CT-ADE-SOC test set.
